# Five new species of the genera *Heerz* Marsh, *Lissopsius* Marsh and *Ondigus* Braet, Barbalho and van Achterberg (Braconidae, Doryctinae) from the Chamela-Cuixmala biosphere reserve in Jalisco, Mexico

**DOI:** 10.3897/zookeys.164.2201

**Published:** 2012-01-11

**Authors:** Alejandro Zaldívar-Riverón, Juan José Martínez, Fadia Sara Ceccarelli, Scott R. Shaw

**Affiliations:** 1 Colección Nacional de Insectos, Instituto de Biología, Universidad Nacional Autónoma de México, 3er. circuito exterior s/n, Cd. Universitaria, Copilco Coyoacán, A. P. 70-233, C. P. 04510., D. F., México; 2 Department of Renewable Resources, University of Wyoming, USA

**Keywords:** new species, *Heerz*, *Lissopsius*, *Ondigus*, 28S, COI

## Abstract

Five new species belonging to the poorly known Neotropical doryctine parasitoid wasps genera *Heerz* Marsh (*Heerz ecmahla* **sp. n.** and *Heerz macrophthalma* **sp. n.**), *Lissopsius* Marsh (*Lissopsius pacificus* **sp. n.** and *Lissopsius jalisciensis* **sp. n.**) and *Ondigus* Braet, Barbalho & van Achterberg (*Ondigus cuixmalensis* **sp. n.**) are described from the Chamela-Cuixmala Biosphere reserve in Jalisco, Mexico. Keys to the described species of the above three genera are provided. The phylogenetic placement of the examined taxa is investigated based on mitochondrial (COI) and nuclear (28S, 2^nd^ and 3^rd^ domain regions) DNA sequence data.

## Introduction

The Doryctinae represents one of the largest subfamilies of braconid parasitoid wasps, probably only behind Braconinae, Microgastrinae, Alysiinae and Opiinae (Jones et al. 2009). Currently, there are almost 200 recognised doryctine genera, of which about 60% have been described from the Neotropical region (Belokobylskij 1992; Belokobylskij et al. 2004; Marsh 2002). The doryctine diversity that occurs north of Costa Rica to central Mexico, however, has been scarcely explored despite that the fauna of this region is known to have high levels of endemism with both Neotropical and Nearctic affinities (e.g. Halffter 1964; Ortega and Arita 1998).

A recent barcoding study carried out in the Chamela-Cuixmala Biosphere Reserve (CCBR) in Jalisco, Mexico, has revealed the existence of an extraordinary, largely neglected doryctine fauna (Zaldívar-Riverón et al. 2010). This reserve is located near the Mexican Pacific coast and is mainly composed by one of the best preserved tropical dry forests in the country (Noguera et al. 2002). Most of the doryctine genera found in this region appear to have Neotropical affinities, with many of them also being reported for Costa Rica (Marsh 2002), although there also are some genera that are mainly from Old World and Nearctic (e.g. *Spathius* Nees, *Caenophanes* Foerster, *Rhaconotus* Ruthe) (Zaldívar-Riverón et al. 2010).

Three new species representing novel records for two doryctine genera in the Mexican territory have so far been described from the CCBR (*Neoheterospilus* Belokobylskij and *Iare* Barbalho & Penteado-Días: Martínez and Zaldívar-Riverón 2010; Martínez et al. 2010). In this work, we describe five new doryctine species from the above region belonging to the rare genera *Heerz* Marsh *Lissopsius* Marsh and *Ondigus* Braet, Barbalho & van Achterberg. The phylogenetic placement of these taxa is also investigated using mitochondrial and nuclear DNA sequence markers. These five species represent new records for their genera in Mexico, and their description becomes relevant since there are plans to build new touristic developments near the CCBR, which is the only known locality for these genera in the country.

## Methods

All the specimens included in this work were collected in several field trips carried out during 2009–2011 to the Chamela Biological station (within the CCBR) owned by the Instituto de Biología, Universidad Nacional Autónoma de México. All the collected specimens were preserved in 100% ethanol, kept at -20°C until they were processed for DNA sequencing, and subsequently dried, labelled and pinned. The material examined in this study is deposited in the Colección Nacional de Insectos, Instituto de Biología, Universidad Nacional Autónoma de México (IB-UNAM CNIN), Museo Argentino de Ciencias Naturales “Bernardino Rivadavia”, Buenos Aires, Argentina (MACN), and the University of Wyoming Insect Museum (UWIM).

The terminology employed follows Sharkey and Wharton (1997), except for the surface sculpture, which follows Harris (1979). Colour photographs were taken and edited with a Leica® Z16 APO-A stereoscopic microscope, a Leica® DFC295/DFC290 HD camera, and the Leica Application Suite® program. Digital SEM photographs were taken with a FEI INSPECT (5350 NE Dawson Creek Drive Hillsboro, Oregon 97124, USA) SEM) in low vacuum at the Museo Nacional de Ciencias Naturales (CSIC, Madrid, Spain).

### Phylogenetic placement of new taxa

The phylogenetic placement of the new taxa described in this study was reconstructed based on two widely used gene markers, around 658 bp of the barcoding locus [cytochrome oxidase I (COI) mitochondrial (mt) DNA gene], and a ~650 bp fragment corresponding to the second and third domain regions of the nuclear 28S rDNA gene. For the specimens belonging to *Ondigus*, a single leg was removed, placed in a 96-well lysis plate and posted to the University of Guelph for DNA extraction, amplification and sequencing (see laboratory protocols in Smith et al. 2009). DNA extraction and PCR products of the two markers for the specimens belonging to *Heerz* and *Lissopsius* were obtained at IB-UNAM following the protocols described in Ceccarelli et al. (2012). Non-purified PCR products were subsequently sent to the High-Throughput Genomics Unit at the University of Washington (http://www.htseq.org/index.html) for DNA sequencing. The COI and 28S primers employed were LepF1/LepRI (Hebert et al. 2004) (LEP-F1: 5’-ATT CAA CCA ATC ATA AAG ATA T-3’; LEP-R1: 5’-TAA ACT TCT GGA TGT CCA AAA A-3’) and 28S-FD2 (Belshaw and Quicke 1997) (fwd: 5’ GCG AAC AAG TAC CGT GAG GG 3’) 28SRD3 (Mardulyn and Whitfield 1999) (rev: 5’ TAG TTC ACC ATC TTT CGG GTC CC 3’), respectively. All sequences were edited with Sequencher version 4.0.5 (Gene Codes).

All the sequences generated for this study are deposited in GenBank (see accession numbers below). These sequences will be also available in the project file ‘Parasitoid Wasps (Braconidae: Doryctinae) of Chamela-Cuixmala Biosphere Reserve’ (ASDOR project) in the projects section of the Barcode of Life Data System (www.barcodinglife.org).

Genetic distances of the COI marker within and among the newly described taxa examined were calculated using the K2Pdistance model (Kimura 1980) with PAUP version 4.0b10 (Swofford 2002). The phylogenetic placement of these taxawithin the Doryctinae was reconstructed using a previously published COI+28S matrix containing 64 doryctine genera (94 species) and 21 outgroups belonging to 12 different cyclostome subfamilies (Zaldivar-Riverón et al. 2008). We also included in the above matrix published sequences of specimens belonging to *Callihormius* Ashmead, *Iare* and *Panama* Marsh (Martínez et al. 2010; GenBank accession nos HQ535818-20, HQ535830-32). These and the newly generated COI and 28S sequences were included in the published data set, excising the ambiguously aligned regions for the 28S marker.

We ran a Bayesian MCMC partitioned analysis with Mrbayes version 3.1.2 (Ronquist and Huelsenbeck 2003) through the University of Oslo bioportal (http://www.bioportal.uio.no/). This analysis consisted of two simultaneous runs of 20 million generations each using four chains and default priors. We considered four different partitions, one for the 28S data set and three for COI according to its first, second and third codon positions. The appropriate evolutionary model chosen for each partition was selected following the akaike information criterion obtained with MrModeltest version 2.3 (Nylander 2004) and PAUP version 4.0b10 (Swofford 2002). We excised the first 10 milion sampled trees of each run as a conservative measure for securing stationarity and pooled the remaining trees for reconstructing a majority consensus phylogram with posterior probability (PP) of clades, considering values ≥ 0.95 as significantly supported (Huelsenbeck and Ronquist 2001).

## Results and discussion

### Genetic divergences and phylogenetic placement of new taxa

Genetic distances of the COI marker among the three examined species of *Heerz* using the K2P distance model ranged from 11.1 to 14.6%. Within *Heerz ecmahla* sp. n., the COI sequence fragment varied from 0.18 to 0.37%, whereas a unique haplotype was found among the three sequenced specimens of *Heerz macrophthalma* sp. n. *Lissopsius pacificus* sp. n. and *Lissopsius jaliscoensis* sp. n. also each had unique haplotypes, with a sequence divergence of 8% between them. The COI distance between the sequenced specimens of *Ondigus cuixmalensis* sp. n. and *Ondigus bicolor* Braet, Barbalho & van Achterberg was of 11.4%.

**Figure 1.**
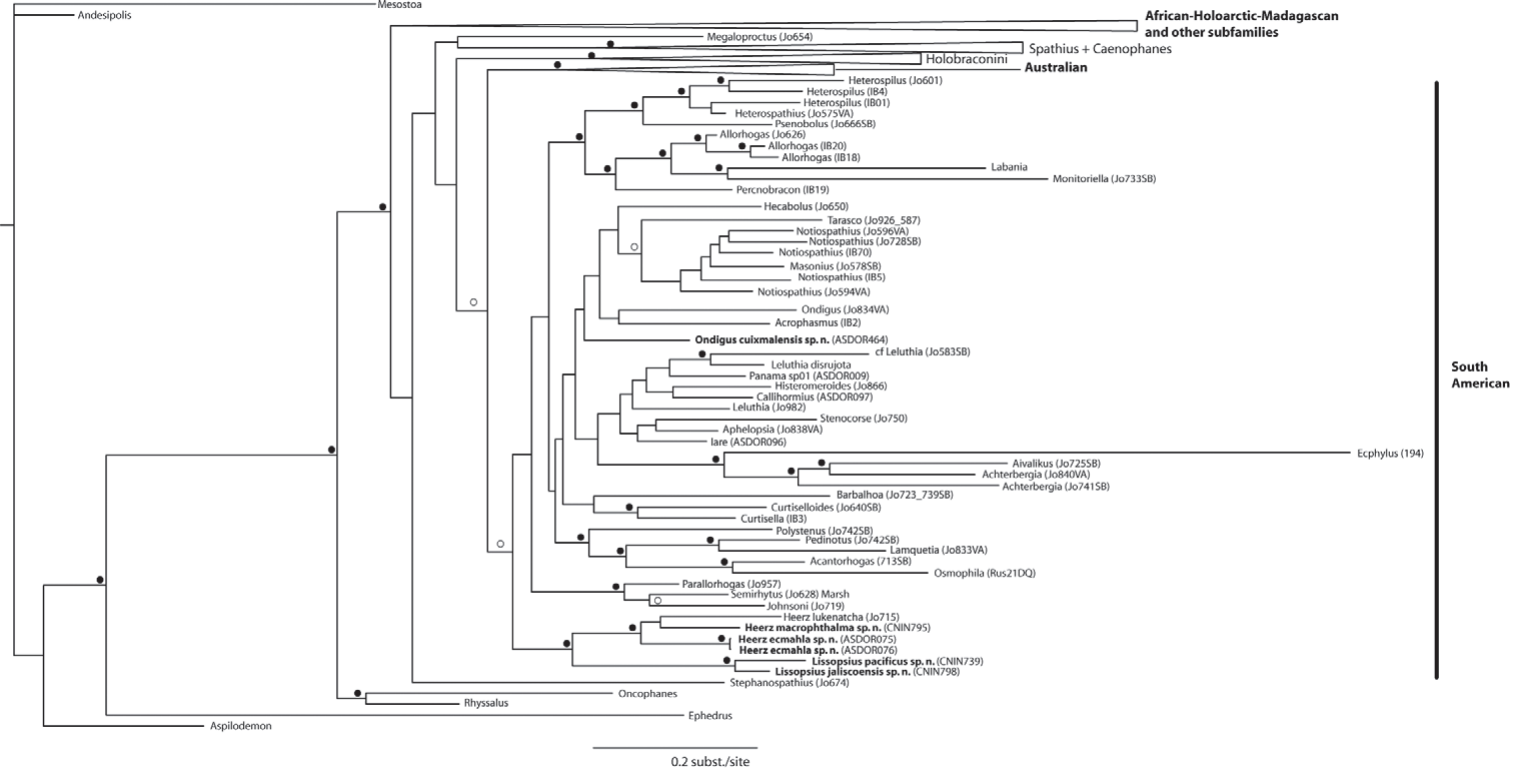
Bayesian phylogram showing the phylogenetic placement of the taxa described in this study within the Doryctinae. Black circles near branches represent posterior probabilities ≥ 0.95; blank circles represent posterior probabilities between 0.90 and 0.94. Names of the major clades are according to Zaldívar-Riverón et al. (2008).

Our reconstructed Bayesian phylogram based on the concatenated 28S+COI data sets (Fig. 1) recovered the species of the three genera examined in this study within a major ‘South American’ clade (PP = 0.93) also recovered in previous phylogenetic studies of the Doryctinae (Zaldívar-Riverón et al. 2007, 2008). Within this South American clade, *Lissopsius* and *Heerz* were each significantly supported as monophyletic (PP = 1.0 in both cases), and were significantly supported as sister taxa (PP = 1.0). Within *Heerz*, *Heerz ecmahla* sp. n. was weakly supported (PP = 0.6) as sister taxon of the *Heerz lukenatcha* Marsh + *Heerz macrophthalma* sp. n. clade. The specimen assigned to *Ondigus cuixmalensis* sp. n. was on the other hand recovered at the base of a group containing a *Notiospathius* Matthews & Marsh + *Masonius* Marsh + *Tarasco* Marsh + *Hecabolus* Curtis and an *Acrophasmus* Enderlein + *Ondigus bicolor* clades, though with a non-significant support (PP = 0.3).

## Taxonomy

### Heerz

Marsh

http://species-id.net/wiki/Heerz

Heerz Marsh, 1993: 17; Marsh 2002: 102.

#### Type species.

*Heerz lukenatcha* Marsh

#### Diagnosis.

**Figure 2.**
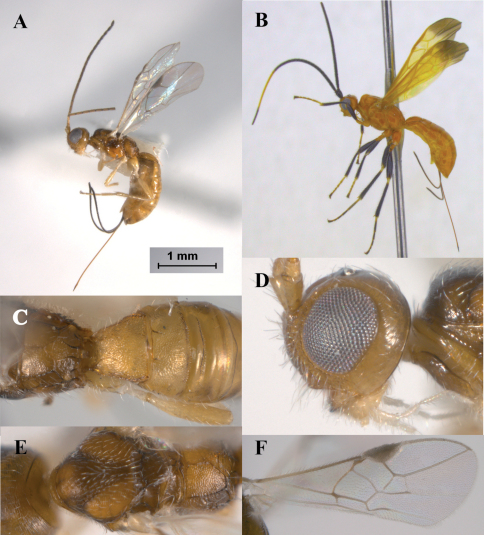
*Heerz ecmhala* sp. n. (holotype) (**A, C–F**) and *Heerz lukenatcha* Marsh (**B**): **A, B** habitus, lateral view **C** propodeum and basal half of metasoma, dorsal view **D** head, lateral view **E** mesosoma, dorsal view **F** fore wing.

**Figure 3.**
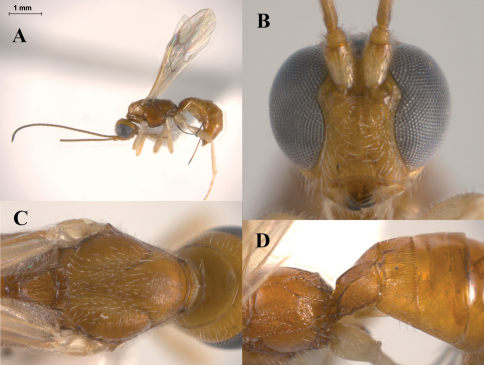
*Heerz macrophthalma* sp. n. (holotype): **A** habitus, lateral view **B** head, frontal view **C** mesosoma, dorsal view **D** propodeum and basal half of mesosoma, dorsolateral view.

**Figure 4.**
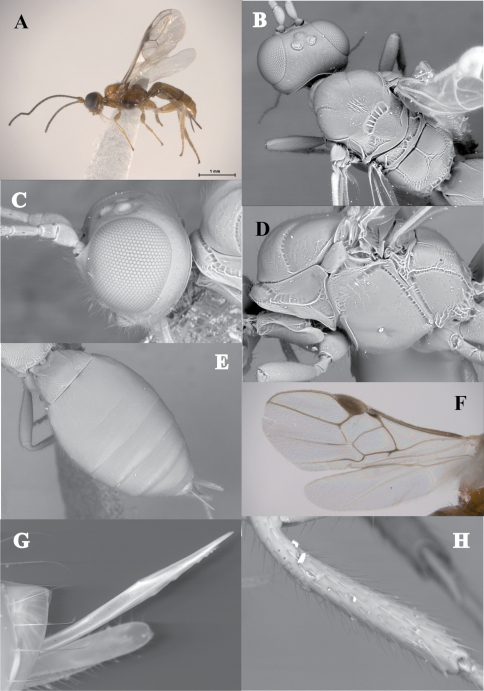
*Lissopsius pacificus* sp. n. (holotype): **A** habitus, lateral view **B** head and mesosoma, dorsal view **C** head, lateral view **D** mesosoma, lateral view **E** metasoma, dorsal view **F** fore and hind wing **G** ovipositor **H** fore tibia.

**Figure 5.**
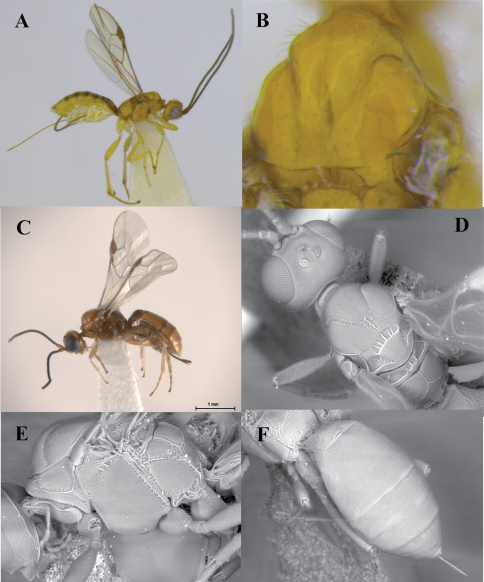
*Lissopsius flavus*Marsh: **A** habitus, lateral view **B** mesoscutum, dorsal view. *Lissopsius pacificus* sp. n. (holotype): **C** habitus, lateral view **D** head and mesosoma, dorsal view **E** mesosoma, lateral view **F** metasoma, dorsal view.

*Heerz* distinguishes from other doryctine genera by the following combination of features: (1) frons excavated (Fig. 2D), (2) propodeum with a longitudinal median carina followed by a pentagonal areola (Figs 2C, 3D), (3) second metasomal tergite entirely or partially sculptured, contrasting with third one, which is smooth, polished and with a transverse furrow (Figs 2C, 3D), (4) vein r-m of fore wing present (Fig. 2F), (5) vein M+CU of hind about as long as vein 1M, (6) vein cu-a of hind wing straight or slightly curved apically towards wing apex, (7) male hind wing without pterostigma, and (8) hind coxa with a distinct basoventral tooth. Species of *Heerz* are very similar in habitus to those of *Lissopsius*, all having a body mostly smooth and shiny, propodeum with a longitudinal median carina followed by a pentagonal areola, and vein M+CU of hind wing slightly shorter to larger than vein 1M. However, *Heerz* differs from *Lissopsius* by having the vein r-m of fore wing present (Fig. 2F) (absent in *Lissopsius*), hind coxa with a basoventral tooth (absent in *Lissopsius*), and ovipositor distinctly sclerotised apically (uniformly slcerotised in *Lissopsius*).

#### Description.

Small to moderate size, 2.5-7.0 mm; eyes large, moderately to distinctly emarginated opposite antennal sockets; frons concave; occipital carina present, meeting hypostomal carina before mandible; labrum distinctly concave; hypoclypeal depression small and round; clypeus short; malar suture absent; maxillary palpi 5-segmented, labial palpi 4-segmented; head and mesosoma smooth or weakly sculptured; mesoscutum declivous anteriorly; prepectal carinae present; precoxal sulcus smooth; surface of propodeum smooth on anterior half, slightly rugose on posterior half, with a median longitudinal carina followed by a pentagonal areola; metapleural flange present; fore tibia with a row of spines along anterior edge; hind coxa with a distinct basoventral tooth; vein m-cu of fore wing antefurcal to vein 2RS, thus (RS+M)b present; vein 1cu-a postfurcal to vein 1M; vein r-m of fore wing present; second submarginal cell distinctly short; first subdiscal cell of fore wing open at apex; vein M+CU of hind wing slightly shorter to larger than vein 1M; males without pterostigma on hind wing; basal sternal plate (acrosternite) of first metasomal tergite short, 0.2-0.3 times the length of tergum; first and second metasomal tergites scupltured; third metasomal tergite smooth with a transverse furrow; remaining metasomal tergites smooth; ovipositor strongly sclerotised apically; nodes reduced, only one or absent.

#### Distribution.

Brazil, Costa Rica and Mexico.

#### Remarks.

The two new species of *Heerz* described below considerably modify the previous concept of the genus. The two previously described species, *Heerz lukenatcha* and *Heerz tooya* Marsh, are characterised by their smooth mesosoma, dusky wings and relatively large body size. The Mexican species, on the other hand, have a coriaceous mesoscutum, uniformly hyaline wings and are considerably smaller, especially *Heerz ecmahla*. Moreover, frons excavation is more conspicuous in the two new species compared to *Heerz lukenatcha* and *Heerz tooya*. Despite these morphological differences, our comparisons with type material and our DNA sequence data (see below) led us to include the new species within *Heerz*.

#### Key to described species of *Heerz* (modified from Marsh 1993)

**Table:** 

1	Wings partially or totally infuscate (Fig. 2B), mesoscutum mostly smooth	2
–	Wings hyaline (Fig. 2A, 3A), mesoscutum coriaceous (Fig. 3C)	3
2	Wings yellow on basal ¾, dusky on apical ¼, all femora and tibiae black	*Heerz lukentacha* Marsh
–	Wings evenly dusky; all femora and tibiae yellow	*Heerz tooya* Marsh
3	Eyes considerably large, their height 5.0 times longer than malar space, inner orbit clearly emarginated (Fig. 3B); second metasomal tergite mostly striate (Fig. 3D); ovipositor 0.5 times as long as metasoma (Fig. 3A)	*Heerz macrophthalma* sp. n.
–	Eyes small, their height about 3.0 times length of malar space (Fig. 2D), weakly emarginated; second metasomal tergite mostly coriaceous (Fig. 2C); ovipositor slightly longer (about 1.1 times) than metasoma (Fig. 2A)	*Heerz ecmahla* sp. n.

### Heerz ecmahla

Martínez, Zaldívar-Riverón, Ceccarelli & Shaw sp. n.

urn:lsid:zoobank.org:act:79D0180F-1C9F-4825-97A6-6E4C3C706CCB

http://species-id.net/wiki/Heerz_ecmahla

Figs 2A–F

#### Diagnosis.

*Heerz ecmahla* distinguishes from the remaining species of the genus by the uniformly coriaceous sculpture on the first and second metasomal tergites (Fig. 2C). It also distinguishes from the Central and South American species, *Heerz tooya* and *Heerz lukenatcha*, by its entirely hyaline wings (Fig. 2A) [wings partially or totally infuscate in the latter two species (Fig. 2B)], and from *Heerz macrophtalma* by its relatively smaller eyes (Fig. 2D) [considerably large in *Heerz macrophtalma* (Fig. 3B)] and distinctly longer ovipositor (Fig. 2A) [about 0.5 times as long as metasoma in *Heerz macrophtalma* (Fig. 3A)].

#### Description.

Female. *Colour*: Body honey yellow, antennae honey yellow, turning darker apically; legs yellow, median lobe of mesoscutum and upper half of mesopleuron light brown; metasoma slightly lighter; wings hyaline, veins, pterostigma and tegula light brown. *Body length*: 2.5 mm. *Head*: vertex, frons and temple striate, gena smooth, face acinose; eyes large, its height 1.3 times its maximum width (lateral view); malar space about 0.3 times eye height (lateral view); ocello-ocular distance two times the diameter of lateral ocellus and 1.5 times longer than posterior ocellar line; antennae with 21 flagellomeres. *Mesosoma*: length of mesosoma 1.9 times its maximum height; pronotum essentially smooth, pronotal groove largely smooth, with a few rugae medially; mesoscutal lobes coriaceous; notauli scrobiculate, obscured in a posterior rugose median area; scutellum smooth, scutellar sulcus deep and scrobiculate, with four longitudinal carinae; mesopleuron largely smooth, posterior mesopleural sulcus distinct and scrobiculate; subalar groove scrobiculate; precoxal sulcus smooth; metapleuron rugulose; dorsolateral areas of propodeum coriaceous; propodeal areola distinctly delimited by carinae and essentially coriaceous. *Legs*: fore tibia with a row of 12 spines; hind coxa mostly smooth, with a distinct basoventral tooth. *Wings*: fore wing 2.9 times longer than wide; length of pterostigma 2.7 times its maximum width, 0.6 times length of vein R; vein r 1.5 times longer than vein 3RSa; vein 3RSa 0.8 times length of r-m; vein m-cu distinctly antefurcal; (RS+M)b present, 0.3 times length of vein 2RS; hind wing with vein M+CU 0.8 times as long as 1M and 1.5 times length of vein r-m; vein m-cu slightly curved towards wing apex. *Metasoma*: first metasomal tergite short, 1.1 times as long as its apical width, entirely coriaceous, with two dorsolateral carinae running through anterior half of median tergite, without a median dorsal area fully delimited by carinae; second metasomal tergite coriaceous, with two slightly convergent furrows basally; third metasomal tergite smooth, turning coriaceous laterally and with a transverse scrobiculate furrow; remaining metasomal tergites smooth and polished; ovipositor 1.1 times length of metasoma, with a single, reduced node.

Male. Essentially as female, body length 2.7 mm.

Variation. Females: Body length 2.5–2.7 mm; eyes 1.1–1.2 times higher than wide (lateral view); fore wing length 2.5–2.6 times its maximum width; length of pterostigma 2.6–2.7 times its maximum width; hind wing vein M+CU 0.8–1.0 times as long as vein 1M.

Holotype. IB-UNAM CNIN. Female. Mexico, Jalisco, Estación Biológica Chamela, cerca del Laboratorio, 19.49N, -105.04E, 23–24.vi.2009, 95 msnm, light trap, selva baja caducifolia, H. Clebsch, A. Zaldívar, A. Polaszek col., DNA voucher no. ASDOR076 (CHAM-076), GenBank accession nos JF912210, HQ200616 (IB-UNAM CNIN).

Paratypes. IB-UNAM CNIN, MACN. Two specimens. One female, Mexico, Jalisco, Estación Biológica Chamela, cerca del laboratorio, 19.49N, -105.04E, 23–24.vi.2009, 95msnm, light trap, selva baja caducifolia, H. Clebsch, A. Zaldívar, A. Polaszek col., DNA voucher no. CHAM-075, GenBank accession nos JF912209, HQ200615; one male, Mexico, Jalisco, Estación Biológica Chamela, cerca del laboratorio, 19.49N, -105.04E, 05.v.2011, 99–122 msnm, light trap, selva baja caducifolia, A. Zaldívar, S. Zaragoza, A. Ibarra col., DNA voucher nos CNIN796, ASDOR076 (CHAM-076), GenBank accession nos JQ268746, JF912210, HQ200616.

#### Etymology.

The specific epithet is an anagram of Chamela, the type locality of this species.

### Heerz macrophthalma

Martínez, Zaldívar-Riverón, Ceccarelli & Shaw sp. n.

urn:lsid:zoobank.org:act:2F240FAD-E0C2-43FD-ACBC-B66E432EA6B2

http://species-id.net/wiki/Heerz_macrophthalma

Figs 3A–D

#### Diagnosis.

*Heerz macrophthalma* distinguishes from the remaining species of the genus by its considerably larger eyes (Fig. 3B). It can also be distinguished from the Central and South American species, *Heerz tooya* and *Heerz lukenatcha*, by its entirely hyaline wings (Fig. 3A) [wings partially or totally infuscate in the latter two species (Fig. 2B)] and from *Heerz ecmahla* by the striate sculpture on the second metasomal tergite (Fig. 3D) [coriaceous in *Heerz ecmahla* (Fig. 2C)].

#### Description.

Female. *Colour*: Body mostly honey yellow, antennae honey yellow, turning darker apically; legs creamish white, with apex of tarsomeres darker; wings hyaline, veins, pterostigma and tegula light brown. *Body length*: 4.7 mm. *Head*: vertex, frons and temple striate, face acinose; eyes large, 1.3 times higher than wide (lateral view); malar space about 0.2 times eye height; ocello-ocular distance less than 0.8 times diameter of lateral ocellus and as long as posterior ocellar line; antennae with 31 flagellomeres. *Mesosoma*: length of mesosoma 1.8 times its maximum height; pronotum smooth, pronotal groove scrobiculate; mesoscutal lobes coriaceous; notauli deep and scrobiculate, obscured in a posterior striate-rugose median area; scutellum smooth, scutellar sulcus deep and scrobiculate, with six longitudinal carinae; mesopleuron largely smooth, striate near subalar furrow; posterior mesopleural sulcus distinct and scrobiculate; subalar groove scrobiculate; precoxal sulcus smooth; metapleuron smooth; propodeal areola distinctly delimited by carinae and with crossing transversal rugae. *Legs*: fore tibia with a row of 15 spines; hind coxa striate dorsally, smooth ventrally, with a distinct basoventral tooth. *Wings*: fore wing 2.8 times longer than wide; pterostigma 0.6 times the length of vein R; vein r 1.5 times longer than vein 3RSa; vein 3RSa 0.8 times as long as vein r-m; vein m-cu distinctly antefurcal, vein (Rs+M)b present, 0.2 times length of vein 2RS; hind wing with vein M+CU as long as vein 1M and twice length of vein r-m; vein m-cu slightly curved towards wing apex. *Metasoma*: first metasomal tergite short, about as long as its apical width, with median dorsal area delimited by two dorsolateral carinae, median area rugulose-coriaceous, lateral areas striate; second metasomal tergite striate medially, with coriaceous sculpture in between striations, with two more conspicuous slightly convergent furrows; third metasomal terigte smooth, turning coriaceous laterally, with a transverse scrobiculate furrow; remaining metasomal tergites smooth and polished; ovipositor 0.5 times length of metasoma, without distinct nodes.

Male. Smaller than female, body length 2.7–2.8 mm.

Variation. Female: Body length 4.1–4.6 mm; eyes 1.3–1.4 times higher than wide (lateral view); malar space 0.1–0.2 times eye height (lateral view); ocello-ocular distance 0.7-0.8 times diameter of lateral ocellus; antennae with 28–29 flagellomeres; fore wing length 2.8–2.9 times its maximum width; length of pterostigma 2.6–2.8 times its maximum width; hind wing vein M+CU 1.0–1.1 times longer than vein 1M.

Holotype. IB-UNAM CNIN. Female. Mexico, Jalisco, Estación Biológica Chamela, cerca laboratorio, 19.49, -105.04, 5.v.2011, 99-122 msnm, light trap, selva baja caducifolia, Cham-084, Zaldívar, Zaragoza, Ibarra col. DNA voucher no. CNIN795, GenBank accession nos JQ268745, JQ268749 (IB-UNAM CNIN).

Paratypes. IB-UNAM CNIN, MACN, UWIM. Three specimens. One female, Mexico, Jalisco, Estación Biológica Chamela, camino Búho, 19.49/19.49 N, -105.04/-105.04 E, 25.ii.2010, 106 msnm, sweeping net, selva baja caducifolia, A. Zaldívar col., DNA voucher no. ASDOR761, GenBank accession no. HQ200977; two males, Mexico, Jalisco, Estación Biológica Chamela, cerca laboratorio, 19.49 N, -105.04 E, 20.ii.2011, 99-122 msnm, light trap, selva baja caducifolia, A. Zaldívar, col., DNA voucher nos ASDOR551 (Cham-500), ASDOR555 (Cham-504), GenBank accession nos HQ200979, HQ200967 (IB-UNAM CNIN).

#### Etymology.

The specific epithet derives from the greek words *makros* and *ophthalmos*, in reference to the very large compound eyes of this species.

### Lissopsius

Marsh

http://species-id.net/wiki/Lissopsius

Lissopsius Marsh, 2002: 128.

#### Type species.

*Lissopsius flavus* Marsh, 2002.

#### Diagnosis.

**Figure 6.**
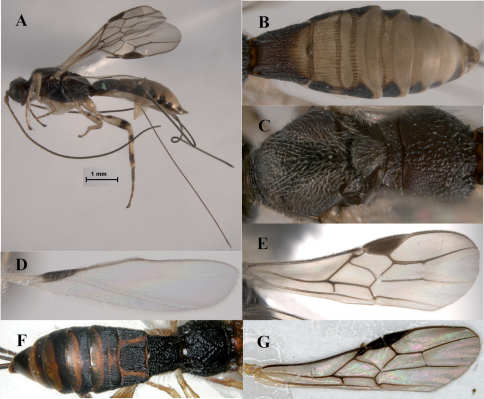
*Ondigus cuixmalensis* sp. n. (holotype): **A** habitus, lateral view **B** metasoma, dorsal view **C **mesosoma, dorsal view **D** hind wing, lateral view (male, paratype) **E** fore wing, lateral view. *Ondigus bicolor* Braet, Barbalho & van Achterberg: **F** propodeum and metasoma, dorsal view **G** fore wing.

This genus distinguishes from other recognised doryctine genera by the following combination of features: (1) body mostly smooth and polished (Figs 4A-E, 5B-F), (2) propodeum with a median longitudinal carina followed by a pentagonal areola (Figs 4B, 5D), (3) vein r-m of fore wing absent (Fig. 4F), (4) vein M+CU of hind wing larger than vein 1M (Fig. 4F), (5) vein cu-a of hind wing curved at apex towards wing tip (Fig. 4F), (6) hind coxa angled at base, without distinct tubercle or tooth, and (7) ovipositor uniformly sclerotised and with a single nodus (Fig. 4G). *Lissopsius* is closely related to *Heerz* (see above) and both are morphologically similar, with a body mostly smooth and polished, propodeal areola present and vein M+CU of hind wing slightly shorter to longer than vein 1M. However, *Lissopsius* differs from *Heerz* by having the vein r-m of fore wing absent (always present in *Heerz*), hind coxa without basoventral tooth (present in *Heerz*), and ovipositor uniformly sclerotised (strongly sclerotised at apex in *Heerz*).

#### Description.

Small size, 2.3-4.5 mm; eyes large, emarginated opposite antennal sockets; occipital carina present, ending before reaching hypostomal carina; labrum distinctly concave; hypoclypeal depression small and round; clypeus short; malar suture absent; maxillary palpi 5-segmented, labial palpi 4-segmented; head, mesosoma and metasoma mostly smooth and polished; mesoscutum declivous anteriorly; prepectal carinae present; precoxal sulcus shallow and almost indistinct; surface of propodeum smooth on anterior half, slightly rugose on posterior half, with median longitudinal carina anteriorly and pentagonal areola posteriorly; metapleural flange present; fore tibia with a row of at least 10 spines along anterior edge (Fig. 4H); hind coxa angled at base, without basoventral tooth; vein m-cu of fore wing considerably antefurcal to vein 2RS, vein (RS+M)b present; vein 1cu-a considerably postfurcal to vein 1M; vein r-m of fore wing absent; first subdiscal cell of fore wing open at apex; vein M+CU of hind wing equal to or slightly longer than vein 1M; males without stigma-like enlargement on hind wing; basal sternal plate (acrosternite) of first metasomal tergite about 0.25 length of tergite.

#### Distribution.

Costa Rica and Mexico.

#### Remarks.

In their study describing new ovipositor diagnostic features for the subfamily Doryctinae, Quicke et al. (1995) proposed two potential morphological synapomorphies for the group: the presence of a double nodus on the dorsal valve of the ovipositor and a strongly sclerotised apex. These characters, however, despite being present in most doryctines, have apparently been reduced or lost in species of various genera (Quicke et al. 1995), including the two of the species of *Heerz* described here (a single or lack of nodus) and the three described known species of *Lissopsius* (uniformly sclerotized apex). The two new species described below represent the first records of the genus outside Costa Rica, and therefore for the Mexican territory. All the specimens of *Lissopsius* included in this work were only collected with light traps, and most of them were collected during three nights in June 2009 and belong to *Lissopsius pacificus* sp. n. The colour of the body in the sequenced specimens of the two species of *Lissopsius* described here became slightly darker after carrying out their DNA extractions, since we used the whole individuals and a non-destructive DNA extraction technique.

#### Key to described species of *Lissopsius*.

**Table:** 

1	Ovipositor considerably short, no more than 0.25 times as long as metasoma (Figs 4A,G, 5C,F); mesoscutum with triangular, longitudinally rugose area in posteromedian area (Figs 4B, 5D)	2
–	Ovipositor long, slightly shorter than metasoma; mesocutum entirely smooth (Fig. 5B)	*Lissopsius flavus* Marsh
2	Second metasomal tergite mostly smooth, only slightly costate basally (Fig. 4E); ventral part of mesopleuron, precoxal sulcus and venter of mesosoma darker that the rest of the body (Fig. 4A); notauli ending before first half of mesoscutum (Fig. 4B)	*Lissopsius pacificus* n. sp.
–	Second metasomal tergite distinctly costate basomedially, remaining area smooth (Fig. 5F); body uniformly yellow (Fig. 5C); notauli ending after first half of mesoscutum (Fig. 5D)	*Lissopsius jaliscoensis* n. sp.

### Lissopsius pacificus

Zaldívar-Riverón, Martínez, Ceccarelli & Shaw sp. n.

urn:lsid:zoobank.org:act:0DDA9D93-E265-40B9-A525-7E185502A6EB

http://species-id.net/wiki/Lissopsius_pacificus

Figs 4 A–H

#### Diagnosis.

This new species distinguishes from *Lissopsius jaliscoensis*sp. n.and *Lissopsius flavus* in having a second metasomal tergite mostly smooth, only slightly costate basally (Fig. 4E) [distinctly costate basally, remaining area smooth in *Lissopsius flavus*; distinctly costate basomedially, remaining area smooth in *Lissopsius jaliscoensis*sp. n.(Fig. 5F)]; ventral part of mesopleuron, precoxal sulcus and venter of mesosoma darker that the rest of the body (Fig. 4A) [mesosoma completely yellow in *Lissopsius flavus* and *Lissopsius jaliscoensis* (Figs 5A,B)]; and notauli ending before first half of mesoscutum (Fig. 4B) [ending after first half of mesoscutum in *Lissopsius flavus* and *Lissopsius jaliscoensis*sp. n. (Figs 5B, D)]. *Lissospius pacificus* also distinguishes from *Lissopsius flavus* in having a very short ovipositor, about 0.25 times as long as metasoma (Fig. 4A,G) [ovipositor as long as metasoma in *Lissopsius flavus* (Fig. 5A)]; a triangular, longitudinally rugose area in the posteromedian area of mesoscutum (Fig. 4B) [smooth in *Lissopsius flavus* (Fig. 5B)]; and smooth third metasomal tergite (Fig. 4E) (third metasomal tergite smooth with weak transverse scrobiculate groove apically in *Lissopsius flavus*).

#### Description.

Female. *Colour*: head yellow, ventral part of mesopleuron, precoxal sulcus and venter of mesosoma light brown, remainder part of mesosoma yellow; metasoma yellow with some areas light brown; pedicel and flagellum yellow to light brown; legs yellow, hind tarsi light brown; wings hyaline, veins and pterostigma brown, tegula yellow. *Body length*: 3.4 mm. *Head*: entirely smooth, vertex and temple pilose, face strongly pilose; eyes large, malar space about 0.2 times eye height; ocello-ocular distance about 1.5 times diameter of lateral ocellus; eye 1.2 times higher than wide (lateral view); antennae broken, with at least 20 flagellomeres; scape with the same length as first flagellomere; first flagellomere longer than second. *Mesosoma*: length of mesosoma 1.7 times its maximum height; pronotum smooth dorsally, slightly rugose ventrally, pronotal groove slightly scrobiculate; mesoscutal lobes smooth, sparsely pilose medially, with a triangular longitudinal rugose area in the posteromedian area of mesoscutum; notauli deep and scrobiculate, not joining, ending before anterior half of mesoscutum; posterolateral sides of scutellum rugose, remaining areas smooth; scutellar sulcus deep and scrobiculate, with five longitudinal carinae; mesopleuron smooth, posterior mesopleural sulcus distinct and scrobiculate, subalar groove puctate; precoxal sulcus shallow and smooth, ending on anterior half of mesopleuron; metapleuron smooth, propodeum smooth on basal half, slightly rugose on apical half, with a median longitudinal carina followed by a distinct pentagonal areola. *Legs*: hind coxa smooth, protruding forward in ventro-anterior corner, about 1.4 times longer than its maximum width. *Wings*: Fore wing length 3.2 times its maximum width, length of pterostigma 2.9 times its maximum width, vein m-cu clearly antefurcal to vein 2RS, vein 1cu-a clearly postfurcal to vein 1M; hind wing vein M+CU 1.4 times longer than vein 1M; vein cu-a curved at apex toward wing tip. *Metasoma*: first metasomal tergite short, about 0.8 times as long as its apical width, medially smooth, laterally slightly costate-punctate; second metasomal tergite slightly costate basally, remaining area smooth; suture between second and third metasomal tergites poorly defined; remaining metasomal tergites smooth and polished; ovipositor very short, about 0.3 times length of metasoma.

Male. Similar to female; body length 2.3–2.8 mm.

Variation. Females: body length 3.0–4.5 mm; eyes 1.1–1.3 times higher than wide (lateral view); malar space 0.1–0.2 times eye height (lateral view); ocello-ocular distance 1.4–1.5 times diameter of lateral ocellus; antennae with 22–26 flagellomeres; fore wing length 2.9–3.2 times its maximum width; length of pterostigma 2.9–3.5 times its maximum width; hind wing vein M+CU 1.4–1.8 times longer than vein 1M.

Holotype. IB-UNAM CNIN. Female. Mexico, Jalisco, Estación de Biología de Chamela, UNAM, camino Búho, 19.49 N, -105.04 E, 65 msnm, 26–27 June 2009, light trap, tropical dry forest, H. Clebsch, A. Zaldívar-Riverón, A. Polaszek col., DNA voucher no. CNIN740, GenBank accession no. JQ268738 (IB-UNAM CNIN).

Paratypes. IB-UNAM CNIN, MACN, UWIM. One hundred and thirteen specimens. Twenty three females, same data as holotype; 37 females, two males, Mexico, Jalisco, Estación de Biológica de Chamela UNAM, camino Búho, 19.49N, -105.04E, 95 msnm, 24–26 June 2009, light trap, tropical dry forest, H. Clebsch, A. Zaldívar, A. Polaszek col.; 49 females, Mexico, Jalisco, Estación Biológica de Chamela, UNAM, cerca del laboratorio, 19.49N, -105.04E, 95 msnm, 23–25 June 2009, light trap, tropical dry forest, H. Clebsch, A. Zaldívar, A.Polaszek col.; four females, Mexico, Jalisco, Estación de Biológica de Chamela, UNAM, camino Chachalaca, 19.49N, -105.03E, 56 msnm, 25 June 2009, light trap, tropical dry forest, H. Clebsch, A. Zaldívar, A. Polaszek col.; one female, Mexico, Jalisco, Estación de Biológica de Chamela, UNAM, near lab, 19.49N, -105.04E, 99 msnm, 5 May 2011, light trap, tropical dry forest, H. Clebsch, A. Zaldívar, A. Polaszek col. DNA voucher nos CNIN739, 740, 742, 743, GenBank accession nos JQ268737, JQ268739-40, JQ268747 (IB-UNAM CNIN). Additional material: About three hundred of specimens preserved in 100% ethanol and kept at -20°C.

#### Biology.

The two new species of *Lissopsius* described in this study apparently have nocturnal habits, since all their specimens were only collected with light traps. These species appear to be generalist parasitoids of various species of lepidopterans according to an ongoing study (Zaldívar-Riverón et al. in prep.) that is being carried out based on molecular analyses of parasitoid linkages (MAPL; Rougerie et al. 2010).

#### Etymology.

The specific name refers to the area where the species was collected, which is situated near the Mexican Pacific coast.

### Lissopsius jaliscoensis

Zaldívar-Riverón, Martínez, Ceccarelli & Shaw sp. n.

urn:lsid:zoobank.org:act:169B8D3A-CE52-4AE6-BCA0-F1D752F2B28E

http://species-id.net/wiki/Lissopsius_jaliscoensis

Figs 5A

#### Diagnosis.

This species distinguishes from *Lissopsius pacificus* and *Lissopsius flavus* by having the second metasomal tergite distinctly costate basomedially (Fig. 5F) [mostly smooth, only slightly costate basally in *Lissopsius pacificus* (Fig. 4D); distinctly costate basally, remaining area smooth in *Lissopsius flavus*]. *Lissopsius jaliscoensis* is morphologically very similar to *Lissopsius pacificus*, but differs from this species by having the mesosoma entirely yellow (Fig. 5C) [ventral part of mesopleuron, precoxal sulcus and venter of mesosoma darker that the rest of the body in *Lissopsius pacificus* (Fig. 4A)], and notauli ending after first half of mesoscutum (Fig. 5D) [ending before first half of mesoscutum in *Lissopsius pacificus* (Fig. 4B)].

#### Description.

Female. *Colour*: head and mesosoma yellow, metasoma yellow with some areas light brown; pedicel and flagellum yellow to light brown; legs yellow; wings hyaline, veins and pterostigma light brown, tegula yellow. *Body length*: 3.3 mm. *Head*: entirely smooth, vertex and temple pilose, face strongly pilose; eyes large, malar space about 0.2 times eye height; ocello-ocular distance about 2.0 times diameter of lateral ocellus; eye 1.1 times higher than wide (lateral view); antennae broken, with at least 24 flagellomeres; scape with the same length as first flagellomere; first flagellomere longer than second. *Mesosoma*: length of mesosoma about 1.7 times its maximum height; pronotum smooth to slightly rugose dorsally and ventrally, pronotal groove slightly scrobiculate; mesoscutal lobes smooth, sparsely pilose; notauli deep and scrobiculate, joining in a triangularly rugose area at the end of mesoscutum; posterolateral sides of scutellum rugose, remaining areas smooth; scutellar sulcus deep and scrobiculate, with five longitudinal carinae; mesopleuron smooth, posterior mesopleural sulcus narrow and scrobiculate, subalar groove slightly punctate; precoxal sulcus almost indistinct and smooth, ending on anterior half of mesopleuron; metapleuron smooth, propodeum smooth on basal half, slightly rugose on apical half, with a median longitudinal carina followed by a distinct pentagonal areola. *Legs*: hind coxa smooth, protruding forward in ventro-anterior corner, about 1.3 times longer than its maximum width. *Wings*: Fore wing length 3.1 its maximum width, length of pterostigma 3.0 times its maximum width, vein m-cu clearly antefurcal to vein 2RS, vein 1cu-a clearly postfurcal to vein 1M; hind wing vein M+CU 1.8 times longer than vein 1M; vein cu-a curved at apex toward wing tip. *Metasoma*: first metasomal tergite short, 0.8 times as long as its apical width, basomedially smooth, remaining area costate with punctate microsculpture; second metasomal tergite distinctly costate with punctate microsculpture basomedially, remaining area smooth; suture between second and third metasomal tergites almost indistinct; remaining metasomal tergites smooth and polished; ovipositor very short, about 0.3 times length of metasoma.

Male. Similar to female. Body length 3.1 mm. Hind wing without stigma-like enlargement.

Variation. Females: body length 3.1-3.7 mm; eye 1.1–1.3 times higher than wide (lateral view); malar space 0.1–0.2 times eye height (lateral view); ocello-ocular distance 1.5–2.0 times diameter of lateral ocellus; all with antennae broken and less than 20 flagellomeres remaining. *Wings*: fore wing length 2.8–2.9 times its maximum width; length of pterostigma 3.8–4.0 times its maximum width; hind wing vein M+CU 1.6–1.8 times longer than vein 1M.

Holotype. IB-UNAM CNIN. Female. Mexico, Jalisco, Estación Biológica de Chamela, UNAM, camino Búho, 19.49N, -105.04E, 65 msnm, 26–27 June 2009, light trap, tropical dry forest. H. Clebsch, A. Zaldívar, A. Polaszek col,. DNA voucher no. CNIN798, GenBank accession nos JQ268742, JQ268748 (IB-UNAM CNIN).

Paratypes. IB-UNAM CNIN, MACN, UWIM. Four specimens. Three females Same data as holotype; one male, Mexico, Jalisco, Estación Biológica de Chamela, UNAM, camino Búho, 19.49N, -105.04E, 95 msnm, 24–25 June 2009, light trap, tropical dry forest, H. Clebsch, A. Zaldívar, A. Polaszek col. DNA voucher nos CNIN741, CNIN798-800, GenBank accession nos JQ268741-44 (IB-UNAM CNIN).

#### Etymology.

The specific name refers to Jalisco, the Mexican state where the species was found.

#### Remarks.

Only five specimens of this species were collected during all our field trips, contrasting with the approximately 300 specimens collected for *Lissopsius pacificus*.

### Ondigus

Braet, Barbalho & van Achterberg

http://species-id.net/wiki/Ondigus

Ondigus Braet, Barbalho & van Achterberg, 2003: 109–111.

#### Type species.

*Ondigus bicolor* Braet, Barbalho & van Achterberg

#### Diagnosis.

This genus distinguishes from the remaining doryctine genera by having the following combination of features: (1) first six metasomal tergites sculptured, fourth to sixth granulate (Figs 6B,F); (2) suture between the second and third metasomal tergites wide, deep, scrobiculate and slightly sinuate (Figs 6B,F); (3) third metasomal tergite with a deep, scrobiculate transverse groove (Figs 6B,F); (4) first subdiscal cell of fore wing open at apex (Figs 6E,G); (5) hind coxa with a distinct basoventral tooth; hind wing vein M+CU approximately the same length of vein 1M (Figs 6E,G); (6) all femora with subbasal tubercles (Fig. 6A).

#### Description.

Moderate size, female 5.6–6.1 mm, male 4.2 mm; eyes large, moderately emarginated opposite antennal sockets; maxillary palpi 5-segmented, labial palpi 4-segmented; face densely setose; occipital carina present and complete; hypoclypeal depression elliptic; clypeus short and wide; malar suture absent; mesosoma setose and mostly coriaceous; mesoscutum declivous anteriorly; notauli distinct anteriorly, scrobiculate, obscured at half of mesoscutum in a longitudinally rugose area; prepectal carinae present; metapleural flange present; all femora with a subbasal protuberance; fore tibia with a row six to eight spines along anterior edge; hind coxa granulate, pilose, with a basoventral tooth; only known male with pterostigma on hind wing; vein m-cu of fore wing interstitial or slightly antefurcal to vein 2RS; vein 1cu-a considerably postfurcal to vein 1M; vein r-m of fore wing present; second submarginal cell of fore wing long; first subdiscal cell of fore wing open at apex; hind wing vein M+CU equal to or slightly longer than vein 1M; first metasomal tergite about 1.1–1.2 times longer than its apical width; suture between second and third metasomal tergite wide, deep, scrobiculate and slightly sinuate; third metasomal tergite with a deep, scrobiculate transverse groove; basal sternal plate (acrosternite) of first metasomal tergite about 0.2 length of tergum.

#### Distribution.

French Guyana, Mexico.

#### Remarks.

The new species of *Ondigus* described here differs in some of the morphological features originally proposed by Braet et al. (2003) to define the genus. These features include a striate vertex, propodeum without longitudinal median carinae and areola, and second metasomal tergite without a pair of sublateral depressions. The above species and *Ondigus bicolor*, however, share a number of diagnostic features on wing venation, legs and sculpture of metasomal tergites. The majority rule consensus tree derived from the Bayesian analysis performed recovered *Ondigus bicolor* and our new species in separate clades; the relationships involved, however, were weakly supported. Based on the above morphological similarities and the unclear phylogenetic relationships obtained, we have decided to place the new species within *Ondigus* until additional help us to confirm the conspecificity of the taxa involved.

#### Key to described species of *Ondigus*.

**Table:** 

1	Vertex striate, propodeum without a median longitudinal carina and areola, dorsolateral carinae of first metasomal tergite ending before its anterior half; second metasomal tergite without a pair of sublateral depressions	*Ondigus cuixmalensis* sp. n.
–	Vertex smooth; propodeum with a median longitudinal carina and a pentagonal areola; dorsolateral carinae of first metasomal median tergite complete; second metasomal tergite with a pair of sublateral depressions	*Ondigus bicolor* Braet, Barbalho & van Achterberg

### Ondigus cuixmalensis

Zaldívar-Riverón, Martínez, Ceccarelli & Shaw sp. n.

urn:lsid:zoobank.org:act:5A802A1E-8FE2-4880-8BBF-BA3516F403D3

http://species-id.net/wiki/Ondigus_cuixmalensis

Figs 6 A–G

#### Diagnosis.

This species differs from the other described species of the genus, *Ondigus bicolor* by having a vertex striate (smooth in *Ondigus bicolor*), propodeum without a median longitudinal carina and areola (Fig. 6C) [both present in *Ondigus bicolor* (Fig. 6F)], dorsolateral carinae of first metasomal tergite ending before its anterior half (Fig. 6B) [running through the apical end of first metasomal tergite in *Ondigus bicolor* (Fig. 6F)], and second metasomal tergite without a pair of sublateral depressions (Fig. 6B) [present in *Ondigus bicolor* (Fig. 6F)].

#### Description.

Female. *Colour*: head and mesosoma dark brown, eye orbits honey yellow; scape and pedicel brown, flagellomeres brown, turning black to apex; palpi pale yellow; first metasomal tergite dark brown, with a semicircular area yellow apically; remaining metasomal tergites yellow, with brown irregular areas laterally; fore, middle and hind coxae, trochanter and trochantellus pale yellow, femora and tibiae pale yellow with irregular specks medially and apically, tarsi light brown to brown; wings hyaline, veins and pterostigma light brown, tegula pale yellow. *Body length*: 5.6 mm; ovipositor 2.5 mm. *Head*: face, frons and vertex striate, temple and gena smooth; malar space about 0.2 times eye height; occipital carina ending just before reaching hypostomal carina; ocello-ocular distance about the same length than diameter of lateral ocellus; eye 1.2 times higher than wide (lateral view); antennae broken, with at least 31 flagellomeres; scape longer than first flagellomere; first flagellomere longer than second. *Mesosoma*: length of mesosoma about 2.0 times its maximum height; pronotum rugose dorsally and ventrally, pronotal groove scrobiculate; mesoscutal lobes coriaceous, slightly transversally rugose at the edges of notauli; notauli deep and scrobiculate, joining before mesoscutum in a rugose area; scutellar disc coriaceous; scutellar sulcus large, deep and scrobiculate, interrupted by scutellar disc, with at least nine longitudinal carinae on each side; mesopleuron porcate-coriaceous dorsally, coriaceous medially and ventrally; precoxal sulcus complete, wide, deep and scrobiculate; venter of mesopleuron coriaceous; posterior mesopleural sulcus narrow and scrobiculate; metapleuron rugose-areolate with coriaceous microsculpture, propodeum longitudinally rugose with coriaceous microsculpture, with a median longitudinal carina on apical half. *Legs*: hind coxa, femur and tibia coriaceous, about 1.4 times longer than its maximum width. *Wings*: Fore wing length 3.7 times its maximum width, length of pterostigma 2.7 times its maximum width, vein m-cu interstitial to vein 2RS, vein 1cu-a clearly postfurcal to vein 1M; hind wing vein M+CU about the same length as vein 1M. *Metasoma*: first metasomal tergite about the same length as its apical width, costate with rugose microsculpture, with dorsolateral carinae ending before its anterior half; second metasomal tergite costate with rugose microsculpture; third metasomal tergite longitudinally striate with granulose microsculpture, with a deep, wide and scrobiculate transversal groove basally; suture between third and fourth metasomal tergites wide, deep, scrobiculate and sinuate; fourth metasomal tergite granulate with basal longitudinal striae; fifth to seventh metasomal tergites granulate, remaining ones smooth; ovipositor long, about 1.7 times length of metasoma.

Male. Smaller than female, body length 4.2 mm; vertex dark brown, rest of head honey yellow; mesopleuron and dorsal and lateral areas of pronotum dark brown to black, remaining part of mesosoma and basal two thirds of first metasomal tergite brown; 30 flagellomeres (complete); vein m-cu antefurcal to vein 2RS; hind wing with stigma; suture between second and third metasomal tergites straight.

Holotype. IB-UNAM CNIN. Female. Mexico, Jalisco, Estación de Biología de Chamela, UNAM, camino Calandria, 19.50N, -105.03W, 45 m, 3 September 2009, sweeping net, tropical dry forest, Hans Clebsch, Alejandro Zaldívar-Riverón, collectors. DNA voucher no. ASDOR464 (CHAM-368), GenBank accession nos HQ201295, HQ200886 (IB-UNAM CNIN).

Paratype. IB-UNAM CNIN, MACN. One specimen. Male. Mexico, Jalisco, Estación de Biología de Chamela, UNAM, camino Calandria, 19.50N, -105.03W, 45 m, 20 February 2010, light trap, tropical dry forest, Alejandro Zaldívar-Riverón, collector. DNA voucher no. ASDOR514 (CHAM-463), GenBank accession no. HQ201294 (IB-UNAM CNIN).

#### Distribution.

Mexico.

#### Remarks.

The COI sequences generated in this work allowed us to associate the only two collected specimens of *Ondigus cuixmalensis*, one male and one female, as conspecific. This is the first known male for the genus, and is characterised by having a stigma-like enlargement on the hind wing.

#### Etymology.

The specific name refers to the CCBR, where this species was collected.

## Supplementary Material

XML Treatment for Heerz

XML Treatment for Heerz ecmahla

XML Treatment for Heerz macrophthalma

XML Treatment for Lissopsius

XML Treatment for Lissopsius pacificus

XML Treatment for Lissopsius jaliscoensis

XML Treatment for Ondigus

XML Treatment for Ondigus cuixmalensis
